# Deep Eutectic Solvents Based on *N*-Methyltrifluoroacetamide and Lithium Bis(trifluoromethanesulfonyl)imide as New Electrolytes with Low Viscosity and High Ionic Conductivity

**DOI:** 10.3390/ma18092048

**Published:** 2025-04-30

**Authors:** Guihong Lyu, Carsten Korte, Jiangshui Luo

**Affiliations:** 1 Laboratory of Electrolytes and Phase Change Materials, College of Materials Science and Engineering & Engineering Research Center of Alternative Energy Materials & Devices, Ministry of Education, Sichuan University, Chengdu 610065, China; 2022223010024@stu.scu.edu.cn; 2 Institute of Energy Technologies—Electrochemical Process Engineering (IET-4), Forschungszentrum Jülich GmbH, 52425 Jülich, Germany; c.korte@fz-juelich.de

**Keywords:** *N*-methyltrifluoroacetamide, LiTFSI, deep eutectic solvents, thermal stability, viscosity, ionic conductivity

## Abstract

In this work, we present a study on the thermal/transport properties of a novel deep eutectic solvent (DES) obtained by using N-methyltrifluoroacetamide (FNMA) as the hydrogen bond donor (HBD) and lithium bis(trifluoromethanesulfonyl)imide (LiTFSI) as the hydrogen bond acceptor (HBA). The binary phase diagram, thermal stability, flammability, viscosity and ionic conductivity of the as-prepared DESs were investigated at atmospheric pressure. The binary phase diagram shows a range of eutectic molar ratios (*x*_LiTFSI_ = 0.2~0.33), with the lowest deep eutectic temperature of −84 °C. At *x*_LiTFSI_ = 0.2 (i.e., FNMA:LiTFSI = 4:1 and denoted as DES-4:1). The as-prepared DES composition exhibits high thermal stability (onset temperature of weight loss = 78 °C), a low viscosity (*η* = 48.9 mPa s at 25 °C), relatively high ionic conductivity (*σ* = 0.86 mS cm^−1^ at 25 °C) and non-flammability. The transport properties, including ionic conductivity and viscosity, as a function of temperature are in accordance with the Vogel–Fulcher–Tammann (VFT) equations. With increasing molar ratio of HBD vs. HBA, the viscosity decreases, and the ionic conductivity increases at a given temperature between 25 °C and 80 °C. The roughly equal pseudo-activation energies for ion transport and viscous flow in each composition imply a strong coupling of ion transport and viscous flow. Walden plots indicate vehicular transport as the main ion transport mechanism for the DES-4:1 and DES-3:1 compositions; meanwhile, it was confirmed that the ionic conductivity and viscous flow are strictly coupled. The present work is expected to provide strategies for the development of wide-temperature-range and safer electrolytes with low salt concentrations.

## 1. Introduction

Deep eutectic solvents (DESs) have garnered significant attention in recent years as a promising class of solvents due to their unique physicochemical properties, such as low vapor pressure, good thermal and chemical stability, high ionic conductivity, non-flammability and compositional tunability [1,2]. DESs were first proposed by Abbott et al. based on a study on choline chloride/urea mixtures at a molar ratio of 1:2 [3]. DESs are typically formed by mixing two or more components that interact as a hydrogen bond donor (HBD) and a hydrogen bond accepter (HBA), which make the melting point much lower than that of either of its components due to the presence of strong intermolecular interactions, such as hydrogen bonds and Lewis acid–base and van der Waals interactions [4,5]. This characteristic has made DESs attractive in various applications, including supercapacitors, electrochromic devices and batteries [6,7,8]. According to Smith and co-workers, DESs can be categorized into four major groups [9]: type I is composed of a metal salt mixed with an organic salt, such as a quaternary ammonium salt. Type II includes hydrated metal halides instead of the water-free ones in type I. Type III is a mixture of organic salts with hydrogen bond donors, like alcohols or amides without metal cations. Type IV is metal salts mixed with hydrogen bond donors.

DESs based on lithium salts and organic components, such as amides, nitriles, tetramethylurea dimers and organosulfides, have been studied by various researchers [10,11,12,13]. They can be regarded as type IV DESs. The eutectic system is formed by intermolecular (Lewis acid–base) interactions between Li^+^ and O/N atoms and the formation of a hydrogen bond, where the anion of the lithium salt is the hydrogen bond acceptor. The DES based on a mixture of 2,2,2-trifluoroacetamide (TFA) and LiTFSI exhibits a relatively low viscosity of 42.2 mPa s and high ionic conductivity of 1.5 mS cm^−1^ at 30 °C at *x*_LiTFSI_ = 0.25 and was successfully introduced as a stable lithium-ion battery electrolyte [14]. The eutectic point was obtained at *x*_LiTFSI_ = 0.2 without other additives. In addition, the ionic conductivity of 3.49 mS cm^−1^ at 25 °C was reported for the DES based on TFA and LiPF_6_ [15].

**Figure 1:**
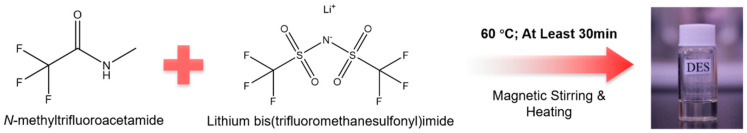
Strategy for preparing the DESs based on FNMA and LiTFSI.

In this work, we report a new transparent DES based on *N*-methyltrifluoroacetamide as the HBD and LiTFSI as the HBA prepared by constant magnetic stirring and heating at 60 °C for at least 0.5 h (Figure 1). The formation of a DES system was analyzed by Fourier transform infrared spectroscopy (FT-IR). Differential scanning calorimetry (DSC) and thermogravimetric analysis (TGA) were conducted to evaluate the thermal properties of as-prepared DESs. In the liquid range of *x*_LiTFSI_ = 0.2~0.33, a eutectic temperature of −84 °C can be determined at *x*_LiTFSI_ = 0.2. The DES-4:1 and DES-3:1 exhibit a low viscosity and relatively high ionic conductivity in the liquid range. The variation of viscosity and ionic conductivity with temperature can be well described by the Vogel–Fulcher–Tammann (VFT) equations. Additionally, its non-flammability is also an advantage for its role as a potential candidate of electrolytes for energy storage systems.

## 2. Experimental

### 2.1. Materials

Lithium bis(trifluoromethanesulfonyl)imide (CAS number: 90076-65-6, abbreviated as LiTFSI, C_2_F_6_LiNO_4_S_2_, ≥98.0%) was obtained from Beijing InnoChem Science & Technology Co., Ltd. (Beijing, China). *N*-Methyltrifluoroacetamide (CAS number: 815-06-5, abbreviated as FNMA, C_3_H_4_F_3_NO, purity ≥98% (GC)) was purchased from Shanghai Aladdin Biochemical Technology Co., Ltd. (Shanghai, China). Both starting materials were used as received.

### 2.2. Preparation of the DESs

Different molar ratios of FNMA (*T*_m_ = 52 °C) and LiTFSI (*T*_m_ = 234 °C), i.e., FNMA:LiTFSI = 1:1, 3:2, 2:1, 3:1, 4:1, 5:1, 6:1 and 7:1, denoted as DES-1:1, DES-3:2, DES-2:1, DES-3:1, DES-4:1, DES-5:1, DES-6:1 and DES-7:1 (for convenience, all of them were denoted as DES) were mixed at room temperature in a sealed glass bottle and heated up to 60 °C with continuous magnetic stirring for at least 0.5 h until a transparent liquid formed (the time can be extended as the sample mass increases). All the starting materials and samples were mixed or stored in a glove box (Mikrouna (Shanghai) Ind. Int. Tech. Co., Ltd., Shanghai, China) with an argon atmosphere (O_2_ and H_2_O contents both lower than 0.01 ppm).

### 2.3. Differential Scanning Calorimetry (DSC)

DSC measurements were conducted between −100 °C and 100 °C using a heat-flux differential scanning calorimeter (STAR^e^ DSC 3, Mettler-Toledo, Greifensee, Switzerland) at a heating and cooling rate of 5 °C min^−1^ to identify the melting point (*T*_m_) and the glass transition temperature (*T*_g_) to explore the phase transition behavior of the as-prepared samples. The heating/cooling cycle was conducted firstly from 25 °C to 100 °C and then from 100 °C to −100 °C, and furthermore from −100 °C to 100 °C with isothermal stabilization for 5 min at each endpoint temperature. To eliminate the possible thermal history, the second run of the DSC cycles was used for analysis. The peak temperature of the melting peak was identified as *T*_m_, and the midpoint of the glass transition as *T*_g_. The nitrogen flow rate during the test was adjusted to 50 mL min^−1^. Each sample mass was controlled to be 5 mg ± 1 mg. Sealed aluminum pans with a volume of 40 μL were used as the crucibles.

### 2.4. Thermogravimetric Analysis (TGA)

The thermal stability of the as-studied samples was evaluated on a thermogravimetric analyzer (TGA 2, Mettler Toledo, Columbus, OH, USA) using an alumina sample pan from 30 °C to 500 °C at a heating rate of 10 °C min^−1^ to identify the weight loss events of the as-prepared samples. The carrier gas was N_2_, with a flow rate of 50 mL min^−1^.

### 2.5. Flammability Test

The flammability of DES-4:1 was tested by direct ignition for 10 s in air via a fire starter in a CR2032 battery case.

### 2.6. Fourier Transform Infrared (FT-IR) Analysis

FT-IR measurements were carried out at room temperature on an FT-IR spectrometer (INVENIO-R, Bruker, Ettlingen, Germany) using an attenuated total reflectance (ATR) attachment. A total of 16 scans with a resolution of 4 cm^−1^ were collected in the region of 4000–400 cm^−1^ for each spectrum. The samples were stored and handled in argon prior to the measurements.

### 2.7. Viscosity

The temperature dependence of the dynamic viscosity of the DESs was measured from 25 °C to 80 °C at a temperature interval of 5 °C, using an Anton Paar Lovis 2000ME viscosimeter (Anton Paar, Graz, Austria) (viscosity reproducibility: <0.1%; temperature accuracy: 0.005 °C).

### 2.8. Density

The temperature dependence of the density of the DESs was measured from 25 °C to 80 °C at a temperature interval of 5 °C, using an Anton Paar DMA 5000 M densitometer (Anton Paar, Graz, Austria) (density reproducibility: 0.000001 g cm^−3^; temperature accuracy: 0.001 °C).

### 2.9. Ionic Conductivity

A conductivity cell was assembled using a CR2032 (Shenzhen Kejing Star Technology Company, Shenzhen, China) coin cell setup with two stainless-steel discs as electrodes and a PTFE gasket as the insulating spacer (inner diameter: 10.0 mm; outer diameter: 16.0 mm; thickness: 1.5 mm). After filling up the inner hole of the PTFE gasket with the liquid sample, the conductivity cell was hermetically sealed in a glove box with argon as the atmosphere. Electrochemical impedance spectroscopy (EIS) was employed in a frequency range from 1 MHz to 1 Hz to measure the ionic conductivity from 25 °C to 80 °C using an electrochemical workstation (Multi Autolab M204, Metrohm Autolab, The Netherlands). The conductivity cell was heated in a thermostat (Viscotemp 15, LAUDA Scientific; temperature measurement stability: ± 0.01 °C) using silicone oil as the heating medium. A thick rubber glove was used for containing the conductivity cell so that the cell was heated indirectly by the silicone oil. A thermocouple was used to measure the temperature directly at the cell. Prior to each measurement, a waiting time of 10 min was kept for thermal equilibrium after reaching the desired temperature. The ionic conductivity (*σ*; unit: S cm^−1^) was calculated using *σ* = *d*/(*R*·*A*), where *R* (Ω) is the resistance determined from the first real axis touchdown point or intercept in the Nyquist plot, *A* (cm^2^) is the area of the gasket hole and *d* (cm) is the thickness of the gasket.

## 3. Results and Discussions

### 3.1. Thermal Properties

**Figure 2:**
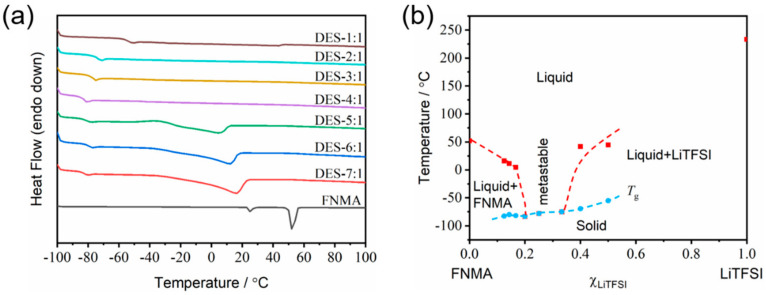
(**a**) DSC traces of the FNMA/LiTFSI system with various molar ratios during the heating scan from −100 to 100 °C, wherein the heating rate is 5 °C min^−1^ and the flow rate of nitrogen gas is 50 mL min^−1^. (**b**) Phase diagram of FNMA-LiTFSI binary system based on the as-prepared DESs with pure FNMA and pure LiTFSI at both ends, wherein *T*_m_ is marked with solid red squares, *T*_g_ with solid blue circles, and the dashed lines are plotted as guidance. The metastable region of *x*_LiTFSI_ = 0.2~0.33 is noted, and the melting points of FNMA (52 °C) and LiTFSI (234 °C) are given.

The thermal properties of the DES electrolytes were first studied by differential scanning calorimetry. The melting points (*T*_m_) of DES-7:1, DES-6:1 and DES-5:1 can be detected as 16 °C, 11 °C and 4 °C, respectively (Figure 2a). With the decreasing molar ratio of FNMA, melting was not observed for the compositions of DES-4:1, DES-3:1 and DES-2:1. Instead, only a low glass transition temperature (*T*_g_) was detected (e.g., −84 °C for DES-4:1), which is a remarkable feature of DESs, indicating that there is a wide range for the liquid phase to exist before decomposition [16]. As the molar ratio of FNMA further decreased, a tiny endothermic peak corresponding to melting could be observed for DES-1:1 (and also DES-3:2) because of the excess LiTFSI. The binary phase diagram as a function of the LiTFSI molar ratio is thus presented in Figure 2b, compiling the measured *T*_m_ and *T*_g_ via the DSC traces for each mixture. The DESs exhibited a typical eutectic character with a broad liquid range for the compositions of *x*_LiTFSI_ = 0.2~0.33. The mixtures turned into a waxy solid (*x*_LiTFSI_ > 0.33). The hydrogen bonding between HBD and HBA is believed to be the underlying reason for the lower melting points of the DESs [17]. The DSC traces and the binary phase diagram prove that a deep eutectic system can be formed between FNMA and LiTFSI with a certain molar ratio.

**Figure 3:**
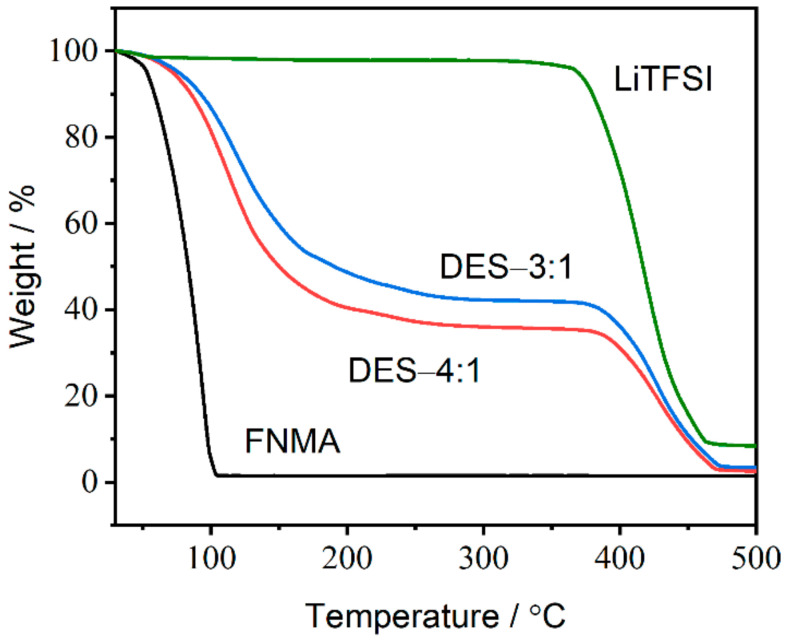
TGA curves of FNMA, LiTFSI, DES-3:1 and DES-4:1. The heating rate was 10 °C min^−1^.

Furthermore, the thermal stability of the starting materials and the as-prepared DESs was characterized by thermogravimetric analysis (TGA). The TGA traces indicate that the onset temperatures (*T*_onset_) of weight loss for pure FNMA and LiTFSI are 52 °C and 381 °C, respectively (Figure 3). However, the TGA traces for DES-4:1 display two weight loss events between 30 °C and 289 °C for the first stage (vaporization of FNMA; *T*_onset_ = 78 °C) and between 336 °C and 481 °C for the second stage (decomposition of LiTFSI; *T*_onset_ = 393 °C), proving that the studied DESs are mixtures of two components. As the LiTFSI ratio of the DESs increased from DES-4:1 to DES-3:1, *T*_onset_ of the weight loss increased from 78 °C to 81 °C for the first stage and from 393 °C to 396 °C for the second stage, respectively. The results demonstrate the higher thermal stability compared to pure FNMA due to the strong intermolecular interactions between FNMA and LiTFSI. The same is observed for DESs based on methyl carbamate (H_2_NCO_2_CH_3_) and lithium salts, which exhibited an increase of 30 °C in the decomposition temperature, indicating the formation of intermolecular interactions [18]. Hence, the decomposition temperature of each DES composition is influenced by the combination of the starting materials and molar ratios of the DESs.

### 3.2. Flammability Test

**Figure 4:**
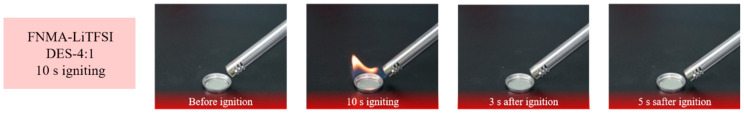
Photographs of flammability test of liquid DES-4:1, which was ignited under open flame for 10 s.

The flammability test (Figure 4) of DES-4:1 indicates that the as-prepared DESs cannot be ignited after continuous combustion in air for 10 s, proving that this composition is non-flammable and offers safety when used as electrolytes in practical applications.

### 3.3. FT-IR Analysis

**Figure 5:**
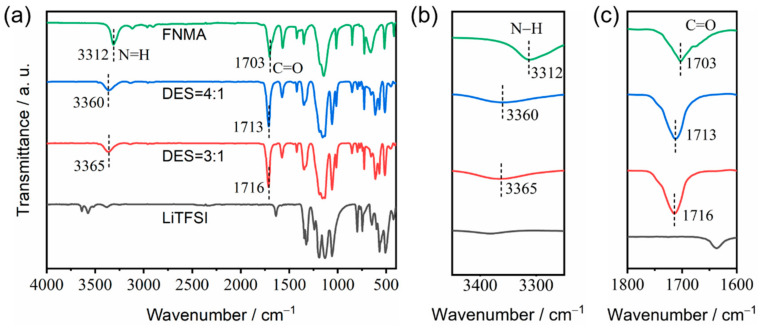
FT-IR spectra of pure FNMA (green line), DES-4:1 (blue line), DES-3:1 (red line) and pure LiTFSI (black line): (**a**) 4000–400 cm^−1^, (**b**) 3450–3250 cm^−1^ and (**c**) 1800–1600 cm^−1^.

Fourier transform infrared (FT-IR) spectroscopy was used to analyze the formation mechanism of the DESs (Figure 5). The strong interaction of *N*-methyltrifluoroacetamide with LiTFSI can promote the dissociation of the lithium ions from anions to form the deep eutectic solution. The bands at 1703 cm^−1^ and 3312 cm^−1^ for *N*-methyltrifluoroacetamide can be attributed to the C=O stretching vibration and the N−H stretching vibration, respectively [18,19]. The DES-4:1 and DES-3:1 compositions both show blue shifts for the C=O and the N−H stretching vibrations. Especially, the C=O stretching vibration shifted to 1713 cm^−1^ (DES-4:1) and 1716 cm^−1^ (DES-3:1), and the N−H stretching vibration shifted to 3360 cm^−1^ (DES-4:1) and 3365 cm^−1^ (DES-3:1), respectively. This may be attributed to the Lewis base–acid interactions of C=O with free Li^+^ and the interactions of N−H with TFSI^−^ anions to form intermolecular hydrogen bonds, weakening the existing N−H···O hydrogen bonds between the FNMA molecules. Therefore, FNMA acts as the hydrogen bond donor and LiTFSI as the hydrogen bond acceptor.

### 3.4. Transport Properties of the As-Prepared DESs

***Ionic conductivity.*** The strong correlation between ionic conductivity (*σ*) and temperature (*T*) was investigated using impedance spectroscopy. The ionic conductivity of DES-4:1 increased from 0.86 mS cm^−1^ to 4.60 mS cm^−1^ as the temperature increased from 25 °C to 80 °C. In comparison, the ionic conductivity of DES-3:1, with an increased content of the lithium salt (i.e., higher salt concentration), was lower than that of DES-4:1. In the same temperature range, it increased from 0.41 mS cm^−1^ to 2.76 mS cm^−1^. It is speculated that the effect of the reduced carrier ion concentration of LiTFSI in DES-4:1 was overcompensated by the reduced viscosity and thus enhanced ion mobility compared with DES-3:1, resulting in higher ionic conductivity of DES-4:1 [20]. While DES-5:1, DES-6:1 and DES-7:1 all have higher melting points (>0 °C) and the components DES-4:1 and DES-3:1 only show glass transition as low as around −84 °C, DES-4:1, with a lower salt concentration (hence, lower cost) and higher ionic conductivity than those of DES-3:1, is the optimized electrolyte component among the binary system of FNMA and LiTFSI in this study.

Generally, the Arrhenius equations (Equation (1)) and the Vogel–Fulcher–Tammann (VFT) equation (Equation (2)) can be used for fitting the data of the ionic conductivity [21,22,23,24]:

(1) $$\sigma =\sigma_{0}\;\exp\left(-\frac{E_{a,\sigma }}{RT}\right)$$

(2) $$\sigma =\sigma_{0}\exp\left(-\frac{B_{\sigma }}{T-T_{0}}\right)$$

where *σ*_0_ (mS cm^−1^), *B*_σ_ (K) and *T*_0_ (K) are the fitting parameters, *T* is the absolute temperature (K), *E*_a,σ_ is the Arrhenius activation energy (kJ mol^−1^), *B*_σ_·*R* is a VFT pseudo-activation energy and *T*_0_ is related to the “ideal” glass transition temperature.

**Figure 6:**
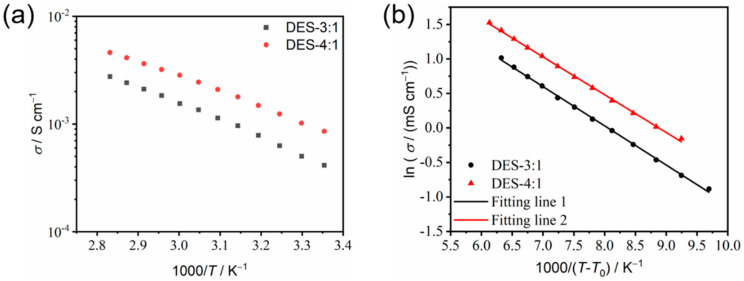
The Arrhenius (**a**) and VFT (**b**) plots of the temperature dependence of the ionic conductivity of DES-3:1 and DES-4:1.

The Arrhenius plot of *σ* as a function of 1000/*T* for the DESs using Equation (1) exhibits a systematic deviation from linearity (Figure 6a), indicating a non-Arrhenian behavior and the significant coupling between viscosity and ionic transport. In contrast, the VFT plot of ln *σ* vs. 1/(*T* − *T*_0_) using Equation (2) shows excellent linearity (Figure 6b). The obtained fitting parameters are presented in Table 1. The values of pseudo-activation energy of DES-3:1 and DES-4:1 are 4.72 kJ mol^−1^ and 4.55 kJ mol^−1^, respectively. The pseudo-activation energy represents the difficulty level of ion movement in solutions; the larger the value, the more difficult it is for the ion to move. The fitting result is consistent with the observed higher ionic conductivity of DES-4:1.

**Figure 7:**
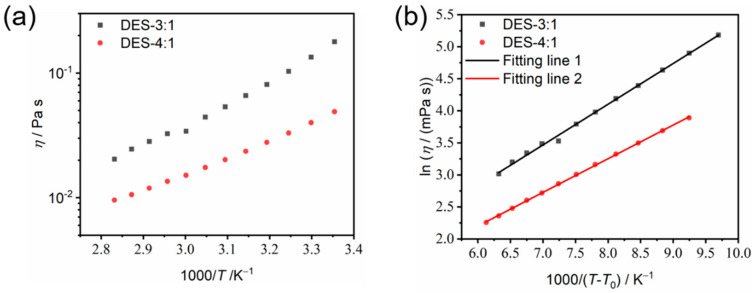
The Arrhenius (**a**) and VFT (**b**) plots of the temperature dependence of the dynamic viscosity of DES-3:1 and DES-4:1.

***Viscosity.*** The dynamic viscosity has a strong influence on the transport properties of a deep eutectic electrolyte solution and is mainly influenced by the strength of hydrogen bonding, molar mass and molar ratios of the HBD and HBA components [25,26], as well as the electrostatic interactions between cations and anions of the constituent salt. Hence, the temperature dependence of the dynamic viscosity of the present DES system was investigated. It was found that from 25 °C to 80 °C, the viscosity decreased from 48.9 mPa s to 9.6 mPa s and from 178.3 mPa s to 20.4 mPa s for DES-4:1 and DES-3:1, respectively (Figure 7a). Interestingly, the dynamic viscosity of DES-4:1 was close to that of propylene glycol at 25 °C. The strong decrease in viscosity with increasing temperature can be attributed to the weakening of intermolecular hydrogen bonding and the electrostatic interactions between the cations and anions due to enhanced thermal motion. In addition, the increase in temperature increases the free volume between molecules, making the molecules flow more easily and the viscosity decrease [27].

The Arrhenius–Andrade equation (Equation (3)) and VFT equation (Equation (4)) are also generally used for the fitting of the dynamic viscosity as a function of temperature [22,23,24,28]:

(3) $$\eta =\eta_{0}\;\exp\left(\frac{E_{a,\eta }}{RT}\right)$$

(4) $$\eta =\eta_{0}\;\exp\left(\frac{B_{\eta }}{T-T_{0}}\right)$$

where *η*_0_ (mPa s), *B*_η_ (K) and *T*_0_ (K) are the fitting parameters, *T* is the absolute temperature (K), *E*_a,η_ is the activation energy (kJ mol^−1^) and *B*_η_·*R* is the pseudo-activation energy (kJ mol^−1^). Again, as the temperature dependence of viscosity deviates from linearity and hence exhibits non-Arrhenian behavior (Figure 7a), the VFT equation is applied. The VFT fitting results can be determined by a linear regression of ln *η* as a function of 1000/(*T* − *T*_0_), as shown in Figure 7b; the results are reported in Table 2 for DES-4:1 and DES-3:1. The obtained activation energy of the temperature dependence of dynamic viscosity for the as-prepared DESs is lower than those of conventional liquids and high-temperature molten salts [29,30].

For the temperature range from 25 °C to 80 °C, the key VFT fitting parameters—pseudo-activation energies (*B*_σ_*R* or *B*_η_*R*) for ion transport and viscous flow in each composition—are roughly equal (*B*_σ_*R* = 4.55 kJ mol^−1^ and *B*_η_*R* = 4.37 kJ mol^−1^ for DES-4:1; *B*_σ_*R* = 4.72 kJ mol^−1^ and *B*_η_*R* = 5.28 kJ mol^−1^ for DES-3:1), indicating a strong coupling of ion transport and viscous flow [31].

**Figure 8:**
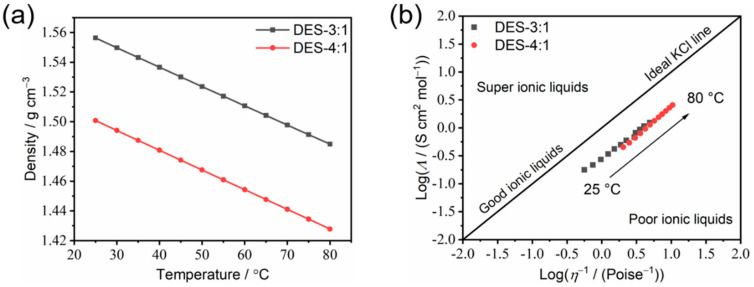
(**a**) The temperature-dependence of density; (**b**) the Walden plots of the DES-3:1 and DES-4:1 compositions from 25 °C to 80 °C. The dynamic viscosity data are taken from Figure 7a. The molar conductivity (*Λ*) is obtained by dividing the ionic conductivity (Figure 6a) by the molar concentration of LiTFSI based on the molar ratio of LiTFSI and the density of each DES (Figure 8a).

Furthermore, by combining the ionic conductivity, density (Figure 8a) and viscosity at different temperatures, the Walden plot for DES-4:1 and DES-3:1, which is a double-logarithmic graph of measured molar conductivities (*Λ*) vs. fluidity (reciprocal of viscosity, 1/*η*), is obtained (Figure 8b). The Walden plot is one of the critical approaches to studying the intrinsic ion transport mechanism in liquids [32], which can be divided into several categories, as follows: good ionic liquids (close to the diagonal), poor ionic liquids (below the diagonal) and super ionic liquids (above the diagonal). The presented plots of DES-3:1 and DES-4:1 are close to the diagonal, which means that the as-prepared electrolytes can be categorized as “good ionic liquid” with good ionicity. This is mainly due to the strong interactions between the starting materials, which triggered a deep eutectic phenomenon, resulting in low viscosity and relatively high ionic conductivity. The slope of the Walden plot, α, which is equal to the ratio of the pseudo-activation energies (*B*_σ_/*B*_η_) 31], was found be around 1 for both DES-4:1 and DES-3:1 (Figure 8b), implying vehicular transport as the main ion transport mechanism for these two compositions and confirming that the ionic conductivity and viscous flow are strictly coupled.

## 4. Conclusions

A series of novel deep eutectic solvents based on the *N*-methyltrifluoroacetamide/LiTFSI system have been prepared and characterized by differential scanning calorimetry, thermogravimetry, infrared spectroscopy, viscosity and ionic conductivity measurements. The DES systems with a molar ratio of LiTFSI (*x*_LiTFSI_) between 0.2 and 0.33 appear as metastable liquid phases over a wide temperature range. When *x*_LiTFSI_ = 0.2 (i.e., the composition DES-4:1) a eutectic temperature of −84 °C can be found. At room temperature, a low viscosity of 48.9 mPa s and high ionic conductivity of 0.86 mS cm^−1^ of DES-4:1 indicate it as the optimal composition. The thermal stability of the DES system is improved compared to pure *N*-methyltrifluoroacetamide. The viscosity and ionic conductivity vs. temperature both obey the Vogel–Fulcher–Tammann (VFT) equation very well. The VFT fitting results and Walden plots reveal that the ionic conductivity and viscous flow are strongly coupled and that vehicular transport is the main ion transport mechanism for the DES-4:1 and DES-3:1 compositions. In summary, the as-studied DES systems exhibit a series of advantages, including a wide liquid temperature range, good thermal stability, low salt concentration, low viscosity, high ionic conductivity and non-flammability. This work is anticipated to offer insights into the development of wide-temperature-range and safer electrolytes with a lower salt concentration and lower cost.

## Figures and Tables

**Table 1:** VFT equation fitting parameters for the ionic conductivity of the as-prepared DESs.

Component	*σ*_0_/mS cm^−1^	*T*_0_/K	*B*_σ_/K	*B*_σ_*R*/kJ mol^−1^	*R* ^2^
DES-3:1	97.51	195	568.25	4.72	0.9987
DES-4:1	130.32	190	547.81	4.55	0.9989

**Table 2:** VFT fitting parameters for the dynamic viscosity of the as-prepared DESs.

Component	*η*_0_/mPa s	*T*_0_/K	*B*_η_/K	*B*_η_*R*/kJ mol^−1^	*R* ^2^
DES-3:1	0.38	195	635.44	5.28	0.9976
DES-4:1	0.39	190	525.36	4.37	0.9996

## Data Availability

The original contributions presented in this study are included in the article. Further inquiries can be directed to the corresponding author.
